# Adherence to response-guided pegylated interferon and ribavirin for people who inject drugs with hepatitis C virus genotype 2/3 infection: the ACTIVATE study

**DOI:** 10.1186/s12879-017-2517-3

**Published:** 2017-06-13

**Authors:** Evan B. Cunningham, Behzad Hajarizadeh, Olav Dalgard, Janaki Amin, Margaret Hellard, Graham R Foster, Philip Bruggmann, Brian Conway, Markus Backmund, Geert Robaeys, Tracy Swan, Philippa S. Marks, Sophie Quiene, Tanya L Applegate, Martin Weltman, David Shaw, Adrian Dunlop, Julie Bruneau, Håvard Midgard, Stefan Bourgeois, Maria Christine Thurnheer, Gregory J Dore, Jason Grebely, on behalf of the ACTIVATE Study Group

**Affiliations:** 1 The Kirby Institute, UNSW Sydney, Sydney, NSW Australia; 2 Akershus University Hospital, Oslo, Norway; 3 The Liver Unit, Queen Mary University of London, London, UK; 4 Arud Centres for Addiction Medicine, Zurich, Switzerland; 5 Vancouver Infectious Diseases Center, Vancouver, BC Canada; 6 Ludwig Maximilians-University Munich, Munich, Germany; 7 Department of Gastroenterology and Hepatology, Ziekenhuis Oost Limburg, Genk, Belgium; 8 Department of Hepatology, UZ Leuven, Leuven, Belgium; 9 UHasselt, Hasselt, Belgium; 10 Treatment Action Group, New York, NY USA; 11 Nepean Hospital, Sydney, NSW Australia; 12 Royal Adelaide Hospital, Adelaide, South Australia Australia; 13 School of Medicine and Public Health, University of Newcastle, Newcastle, NSW Australia; 14 Burnet Institute, Melbourne, VIC Australia; 15 Research Center, Centre Hospitalier de l’Universite de Montreal (CRCHUM), Montreal, Quebec, Canada; 16 Stuivenberg ZNA, Antwerp, Belgium; 17 Department of Infectious Diseases, Bern University Hospital, University of Bern, Bern, Switzerland

**Keywords:** Hepatitis C, Treatment, PWID, Injection drug use, Adherence

## Abstract

**Background:**

The aims of this analysis were to investigate treatment completion and adherence among people with ongoing injecting drug use or receiving opioid substitution therapy (OST) in a study of response-guided therapy for chronic HCV genotypes 2/3 infection.

**Methods:**

ACTIVATE was a multicenter clinical trial recruited between 2012 and 2014. Participants with genotypes 2/3 were treated with directly observed peg-interferon alfa-2b (PEG-IFN) and self-administered ribavirin for 12 (undetectable HCV RNA at week 4) or 24 weeks (detectable HCV RNA at week 4). Outcomes included treatment completion, PEG-IFN adherence, ribavirin adherence, and sustained virological response (SVR, undetectable HCV RNA >12 weeks post-treatment).

**Results:**

Among 93 people treated, 59% had recently injected drugs (past month), 77% were receiving OST and 56% injected drugs during therapy. Overall, 76% completed treatment. Mean on-treatment adherence to PEG-IFN and ribavirin were 98.2% and 94.6%. Overall, 6% of participants missed >1 dose of PEG-IFN and 31% took <95% of their prescribed ribavirin., Higher treatment completion was observed among those receiving 12 vs. 24 weeks of treatment (97% vs. 46%, *P* < 0.001) while the proportion of participants with 95% on-treatment ribavirin adherence was similar between groups (67% vs. 72%, *P* = 0.664). Receiving 12 weeks of therapy was independently associated with treatment completion. No factors were associated with 95% RBV adherence. Neither recent injecting drug use at baseline nor during therapy was associated with treatment completion or adherence to ribavirin. In adjusted analysis, treatment completion was associated with SVR (aOR 23.9, 95% CI 2.9–193.8).

**Conclusions:**

This study demonstrated a high adherence to directly observed PEG-IFN and self-administered ribavirin among people with ongoing injecting drug use or receiving OST. These data also suggest that shortening therapy from 24 to 12 weeks can lead to improved treatment completion. Treatment completion was associated with improved response to therapy.

ACTIVATE trial registration number: NCT01364090 - May 31, 2011

**Electronic supplementary material:**

The online version of this article (doi:10.1186/s12879-017-2517-3) contains supplementary material, which is available to authorized users.

## Background

Among people who inject drugs (PWID), there is a substantial burden of hepatitis C virus (HCV) infection [1, 2]. Adherence [3–6] and treatment completion [3] are associated with sustained virologic response (SVR). Adherence to HCV therapy among PWID is of particular interest given the high cost of new direct-acting antiviral (DAA) therapies and the importance of maximizing the chance of successful therapy.

Adherence refers to the extent to which a person’s behaviour, with respect to timing, dosage and frequency of taking medication, corresponds with agreed recommendations from a healthcare provider [7, 8]. Medication adherence research has been performed in a variety of medical conditions, including diabetes, hypertension, arthritis, pulmonary diseases, HIV and others [9] and on average, patients take 79% of prescribed doses of medications [7]. Adherence to HCV therapy is often defined measuring “80/80 adherence”, or the receipt of ≥80% of scheduled doses for ≥80% of the scheduled treatment period [7]. However, this definition combines the two distinct concepts of treatment completion and missed doses during therapy. Understanding the two concepts individually is important for understanding adherence in this population of PWID.

Among health practitioners, HCV therapy is sometimes withheld from people with ongoing injecting drug use, based on concerns of poor adherence to therapy, and risk of reinfection [10]. However, there are few studies that have evaluated adherence to HCV therapy among people with ongoing injecting drug use. Understanding adherence to therapy among PWID is a key component to the scale-up of DAA therapy among this population.

ACTIVATE is a multicentre international trial evaluating the efficacy of response-guided directly observed pegylated-interferon and self-administered ribavirin therapy for chronic HCV genotypes 2/3 infection among people with ongoing injecting drug use or receiving opioid substitution therapy (OST). Participants with genotypes 2/3 were treated with directly observed peg-interferon alfa-2b and self-administered ribavirin for either 12 weeks (in those with undetectable HCV RNA at week 4, RVR) or 24 weeks (in those with detectable HCV RNA at week 4, no RVR). The primary analysis from this study demonstrated that cirrhosis (vs. no/mild fibrosis [adjusted odds ratio (aOR) 0.33, 95% CI 0.13, 0.86]) predicted reduced SVR, while response at week 4 was associated with increased SVR (aOR 8.11, 95% CI 2.73, 24.10) [11]. While the PEG-IFN based regimen used in this study has been replaced in many settings with new DAA therapies, data on adherence to HCV therapy among PWID is still needed. Given the increased tolerability and simplicity of new DAA regimens compared to PEG-IFN based therapies, results in this study likely represent a lower bound of adherence among PWID using new DAA therapies.

The primary aim of this analysis was to evaluate treatment completion, adherence to therapy and associated factors (including impact of treatment duration) in the ACTIVATE study. Further, this analysis also investigated the effect of treatment completion and on-treatment adherence on response to HCV therapy (as measured by SVR).

## Methods

### Study participants

From May 11 2012, to September 30 2014, participants were enrolled at 17 sites in Australia (*n* = 5), Belgium (*n* = 2), Canada (*n* = 3), Germany (*n* = 1), Norway (*n* = 2), Switzerland (*n* = 3) and the United Kingdom (*n* = 1). The last participant visit was July 15 2015. Study recruitment was conducted through a network of drug and alcohol clinics (*n* = 3), office-based practices (*n* = 2), hospital clinics (*n* = 9), and community clinics (*n* = 3).

Participants had to be more than 18 years of age, have chronic HCV genotype 2 or 3 infection, be HCV treatment-naïve, and have reported recent injecting drug use, defined as injecting drug use within 12 weeks of enrolment. Due to slower than anticipated recruitment, on June 26 2013, a study protocol amendment was implemented to also include people currently receiving OST with no recent injection drug use and people who had injected within 24 weeks prior to enrolment. Participants with HIV infection and decompensated liver disease were excluded. Full eligibility has been previously published [11].

### Study design and intervention

ACTIVATE was an international, multicentre open-label study. Participants received directly observed pegylated interferon alfa-2b (PEG-IFN, 1.5 μg/kg/week) and self-administered ribavirin (RBV, 800–1400 mg daily, weight-based).

Participants with a rapid virological response [RVR, defined as non-quantifiable HCV RNA (<15 IU/ml detected and <15 IU/ml undetected) or undetectable HCV RNA on qualitative assay at week 4] received 12 weeks of therapy (shortened duration). Participants without an RVR [defined as quantifiable HCV RNA (≥15 IU/ml) or detectable HCV RNA on qualitative assay at week 4] received 24 weeks of therapy (standard duration).

### Study assessments

Screening assessments included serum HCV RNA levels, HCV genotype, standard laboratory and clinical testing and self-reported behavioural questionnaires (details have been previously reported [11]).

HCV RNA levels and HCV genotype and subtype were measured as previously described [11]. HCV RNA testing was performed on samples collected at screening, baseline, and weeks 4, 12, 24, 36 and 48 (standard duration). All adverse events were recorded and graded according to a standard scale (details have been previously reported [11]).

Directly observed PEG-IFN adherence was recorded by the study nurse. RBV adherence was determined through the return of unused RBV pills. Self-reported RBV adherence was also measured monthly during study visits while on treatment by a patient-administered questionnaire and was used where returned pill counts were unavailable for that time point.

All participants completed a self-administered questionnaire at enrolment (pre-treatment assessment), at baseline (treatment commencement), every 4th week during treatment, and at 12 and 24 weeks of follow-up. The questionnaires collected information on demographics (age, gender, ethnicity, education level, housing status and history of imprisonment), drug and alcohol use, injecting risk behaviours (injection frequency, use of non-sterile needles, needle and syringe borrowing or lending, and injecting paraphernalia [spoons or mixing containers, drug solution/mix, water or filter] sharing), drug treatment, and symptoms of psychological distress (Depression Anxiety Stress Scale, DASS-21). Stable housing was defined as living in a rented or privately owned house or flat..

Social functioning was measured using the short-form Opioid Treatment Index Social Functioning Scale [12]. Social functioning are scored as a sum of the coded responses with higher scores indicating lower social functioning. Alcohol consumption was evaluated by the Alcohol Use Disorders Identification Test-Consumption (AUDIT-C), derived from the first three questions of the full AUDIT (scores ≥3 and ≥4 indicate hazardous consumption or active alcohol use disorders among women and men, respectively) [13]. Depression was measured using DASS 21 with a depression score of ≥10 indicating depression.

### Study definitions

#### Treatment completion

Participants with no early discontinuation of PEG-IFN/RBV therapy prior to the per-protocol planned end of treatment (12 or 24 weeks for shortened or standard therapy respectively) were defined as having completed treatment. Participants were deemed to have discontinued treatment early if for any reason (e.g. physician advised treatment discontinuation, virological non-response, lost to follow up, patient decision, etc.) a participant did not reach the per protocol defined end of treatment.

#### On-treatment adherence

On-treatment adherence was calculated by determining the number of doses taken as proportion of the expected number of doses during the time on treatment. This measures the proportion of doses received from the time that treatment was initiated until treatment was discontinued or completed.

#### Dose modification

A physician directed increase or reduction in the dose at any time during treatment.

### Study outcomes

The main study outcomes were to assess treatment completion, and on-treatment PEG-IFN and RBV adherence. Evaluation of adherence was based on all participants who received at least one injection of PEG-IFN. Treatment success was defined as undetectable qualitative HCV RNA rates at week 12 (SVR).

### Statistical analysis

Treatment completion and 95% on-treatment adherence (at least 95% of scheduled doses were taken) were assessed. Bi-variate comparisons of characteristics of participants and different measures of adherence across treatment arms were tested using the chi-squared test or Fisher’s exact test as appropriate. Time to treatment discontinuation was evaluated using Kaplan Meier analysis. The impact of treatment completion and on-treatment adherence (both PEG-IFN and ribavirin) on SVR were also evaluated.

Logistic regression analyses were used to estimate crude and adjusted odds ratios (OR) and corresponding 95% confidence intervals (95% CI) to identify predictors of HCV treatment completion and on-treatment ribavirin adherence (at least 95% of scheduled doses were taken). In unadjusted analyses, potential predictors were determined a priori and included sex, age, education, accommodation, employment, current OST treatment, social functioning, current depression, alcohol consumption, injection drug use at baseline (past month), injecting behaviours (frequency and drug injected), and treatment arm. Social functioning was calculated using a validated scale from the Opiate Treatment Index [27] that addresses employment, residential stability, and inter-personal conflict as well as social support. A higher score reflects poorer social functioning. This scale has been validated among opiate users in Australia (range, 0–48) [27]. All variables with *p* < 0.20 in the bivariate analysis were considered for multivariate logistic regression models using a backward stepwise approach, sequentially eliminated and subject to the result of a likelihood ratio test. Statistically significant differences were assessed at *p* < 0.05; *p* values are two-sided. Adjusted models for factors associated with SVR were adjusted for all variables found to be associated with SVR in the primary analysis (fibrosis stage and treatment group) as well as by study site using cluster-robust standard errors [11].

Finally, to determine whether later study visits were associated with RBV adherence, generalized estimating equation (GEE) methods were used. Unadjusted and adjusted GEE models were specified using a gaussian family function. Odds ratios (ORs) with corresponding 95% confidence intervals (CIs) and *p*-values were calculated. All analyses were performed using the statistical package Stata v13.1 (College Station, TX, United States).

## Results

### Participant characteristics

A full description of the participant characteristics is described in the primary paper [11]. In summary, 93 patients were enrolled in the study between May 2012 and August 2014 and initiated PEG-IFN/ribavirin therapy: median age 41, 83% male, 77% on OST, 59% injecting in the past month (Table 1).

**Table 1:** Baseline demographic and clinical characteristics stratified by 12 week and 24 week duration (*n* = 93)

Characteristic, n (%)	Overall (*n* = 93)	12 week (*n* = 61)	24 week (*n* = 26)
Age, median (25%, 75%)	41 (35–49)	41 (34–49)	40 (35–48)
Male sex, n (%)	77 (83)	49 (80)	23 (88)
Drug use in the last 6 months (injecting/non-injecting)	77 (83)	48 (79)	25 (96)
Injecting drug use in the last month	55 (59)	39 (64)	15 (58)
Heroin	33 (35)	23 (37)	10 (38)
Cocaine	10 (11)	7 (11)	3 (12)
Amphetamines	14 (15)	7 (11)	6 (23)
Other opiates	11 (12)	8 (13)	3 (12)
Benzodiazapines	2 (2)	2 (3)	0 (0)
Injecting drug use frequency in the last month			
Never	38 (41)	22 (36)	11 (42)
< daily	40 (43)	29 (48)	10 (38)
> daily	15 (16)	10 (16)	5 (19)
Opioid substitution treatment (ever)	82 (88)	56 (92)	21 (81)
OST and recent injecting (past month) at enrolment			
No OST, recent injecting	30 (32)	20 (33)	9 (33)
OST, no recent injecting	23 (25)	15 (25)	5 (19)
OST, recent injecting	40 (43)	26 (43)	12 (44)
OST and recent injecting (past month) at baseline			
No OST, recent injecting	21 (23)	14 (23)	6 (23)
OST, no recent injecting	34 (37)	21 (34)	8 (31)
OST, recent injecting	38 (41)	26 (43)	12 (46)
Stage of liver disease			
No or mild fibrosis (F0-F1)	63 (68)	44 (72)	16 (62)
Moderate or advanced fibrosis (F2-F3)	20 (22)	12 (20)	5 (19)
Cirrhosis (F4)	10 (11)	5 (8)	5 (19)
Study site distribution			
Europe	38 (41)	24 (39)	13 (50)
Australia	40 (43)	27 (44)	9 (35)
Canada	15 (16)	10 (16)	4 (15)

### PEG-IFN and RBV adherence and early treatment discontinuation

Of the 93 participants who initiated therapy, six participants discontinued therapy before week 4. Reasons for discontinuing therapy prior to week 4 include side effects (*n* = 3), unwillingness to continue treatment (*n* = 1), loss to follow up (*n* = 1), and imprisonment (*n* = 1). Among the remaining 87 participants, 70% (*n* = 61) were HCV RNA undetectable at week 4 (rapid virological response, RVR) and were scheduled to receive 12 weeks of therapy (shortened duration) and 30% (*n* = 26) did not have an RVR at week 4 and were scheduled to receive 24 weeks of therapy (standard duration).

**Fig. 1:**
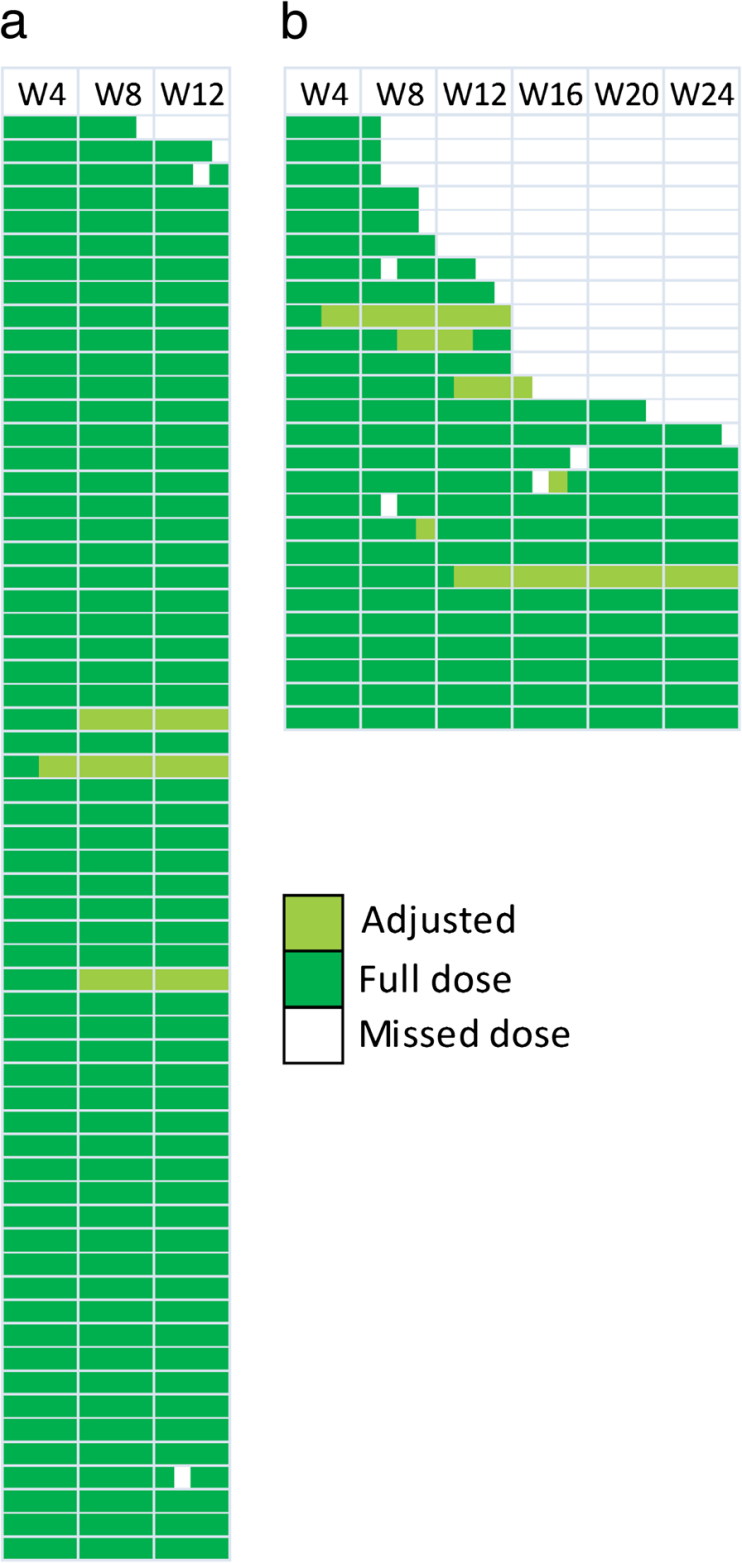
Weekly PEG-IFN adherence among HCV genotype 2/3 participants on shortened (12 weeks; *n* = 61; panel **a**) and standard therapy (24 weeks; *n* = 26; panel **b**). Each row represents a study participant. Dark green boxes represent a full prescribed dose was received, light green boxes represent an adjusted dose was received, and white boxes represent a missed dose

**Fig. 2:**
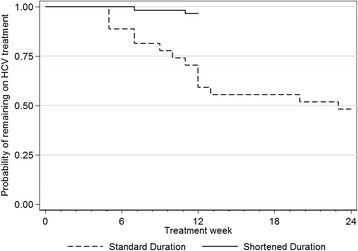
Time to treatment discontinuation among study participants who were in the shortened (12 week; *n* = 61) and standard (24 week; *n* = 26) arm

An abbreviated description of participant adherence is described in the primary paper [11]. Weekly PEG-IFN adherence among those receiving 12 weeks of therapy and 24 weeks of therapy is shown in Fig. 1. Among the entire study population, 76% (*n* = 71) completed HCV therapy (Table 2). Time to discontinuation stratified by duration of therapy is shown in Fig. 2. The median time to discontinuation was 12 weeks and 23 weeks in the 12 week and 24 week groups respectively. Mean on-treatment adherence to PEG-IFN and ribavirin were 98% and 95% (Table 2). Overall, 6% of participants missed >1 dose of PEG-IFN and 31% missed >5% of their doses of ribavirin.

**Table 2:** Adherence to PEG-IFN and RBV among the overall population, those on 12 weeks of therapy, and those on 24 weeks of therapy

Variable	Overall (*n* = 93)	12 week (*n* = 61)	24 week (*n* = 26)
Treatment completion	71 (76)	59 (97)	12 (46)
Mean on-treatment PEG-IFN adherence percent, (SD)	98.2 (4.5)	98.5 (3.1)	98.7 (3.0)
Mean on-treatment ribavirin adherence percent, (SD)	94.6 (8.8)	94.8 (8.1)	94.1 (10.8)
Missed doses of PEG-IFN, n (%)			
No missed doses	87 (94)	59 (97)	22 (85)
1 missed dose	6 (6)	2 (3)	4 (15)
2–5 missed doses	0 (0)	0 (0)	0 (0)
> 5 missed doses	0 (0)	0 (0)	0 (0)
Missed doses of ribavirin, n (%)			
100% of doses taken	22 (25)	16 (26)	5 (20)
95%- < 100% of doses taken	38 (44)	25 (41)	13 (52)
90%- < 95% of doses taken	15 (17)	11 (18)	4 (16)
80%- < 90% of doses taken	7 (8)	6 (10)	1 (4)
< 80% of doses taken	5 (6)	3 (5)	2 (8)
Number of weeks of PEG-IFN therapy, n (%)			
24 weeks	12 (13)	NA	12 (46)
13 to 23 weeks	3 (3)	NA	3 (3)
7 to 12 weeks	69 (74)	61 (100)	8 (31)
0 to 6 weeks	9 (10)	0 (0)	3 (12)
Weeks on PEG-IFN therapy			
Mean, n (SD)	NA	11.9 (0.65)	16.8 (7.8)
Median, n (IQR)	NA	12 (12–12)	21 (10–24)
PEG-IFN dose-modification	9 (10)	3 (5)	6 (23)
Ribavirin dose-modification	21 (23)	12 (20)	9 (35)

Completion of treatment was more frequent in the 12 week group compared to the 24 week group (97% vs. 46%, *P* < 0.001). In the 12 week group, completion of 0–4, 5–8, and 9–12 weeks of therapy was demonstrated by 0% (*n* = 0), 2% (*n* = 1), and 98% (*n* = 60), respectively. In the 24 week arm, completion of 0–4, 5–8, 9–12, 13–23, and 24 weeks was demonstrated by 0% (*n* = 0), 19% (*n* = 5), 23% (*n* = 6), 3% (*n* = 3) and 46% (*n* = 12) respectively. Reasons for discontinuing prior to the planned end of therapy include side effects (overall, *n* = 11; 12 week, *n* = 1; 24 week, *n* = 7), patient unwillingness (overall, *n* = 5; 12 week, *n* = 1; 24 week, *n* = 3), patient lost to follow-up (overall, *n* = 4; 12 week, *n* = 0; 24 week, *n* = 3), and virological failure (overall, *n* = 1; 12 week, *n* = 0; 24 week, *n* = 1). The mean number of weeks of PEG-IFN therapy was 11.9 and 16.8 weeks in the 12 week and 24 week groups respectively.

The mean on-treatment PEG-IFN adherence was similar between those receiving 12 and 24 weeks of therapy (98.5% vs. 98.7%, *P* = 0.811). The proportion of participants missing at least one dose of PEG-IFN while on therapy was 3% in those receiving 12 weeks of therapy compared to 15% in those receiving 24 weeks of therapy (*P =* 0.072).

**Fig. 3:**
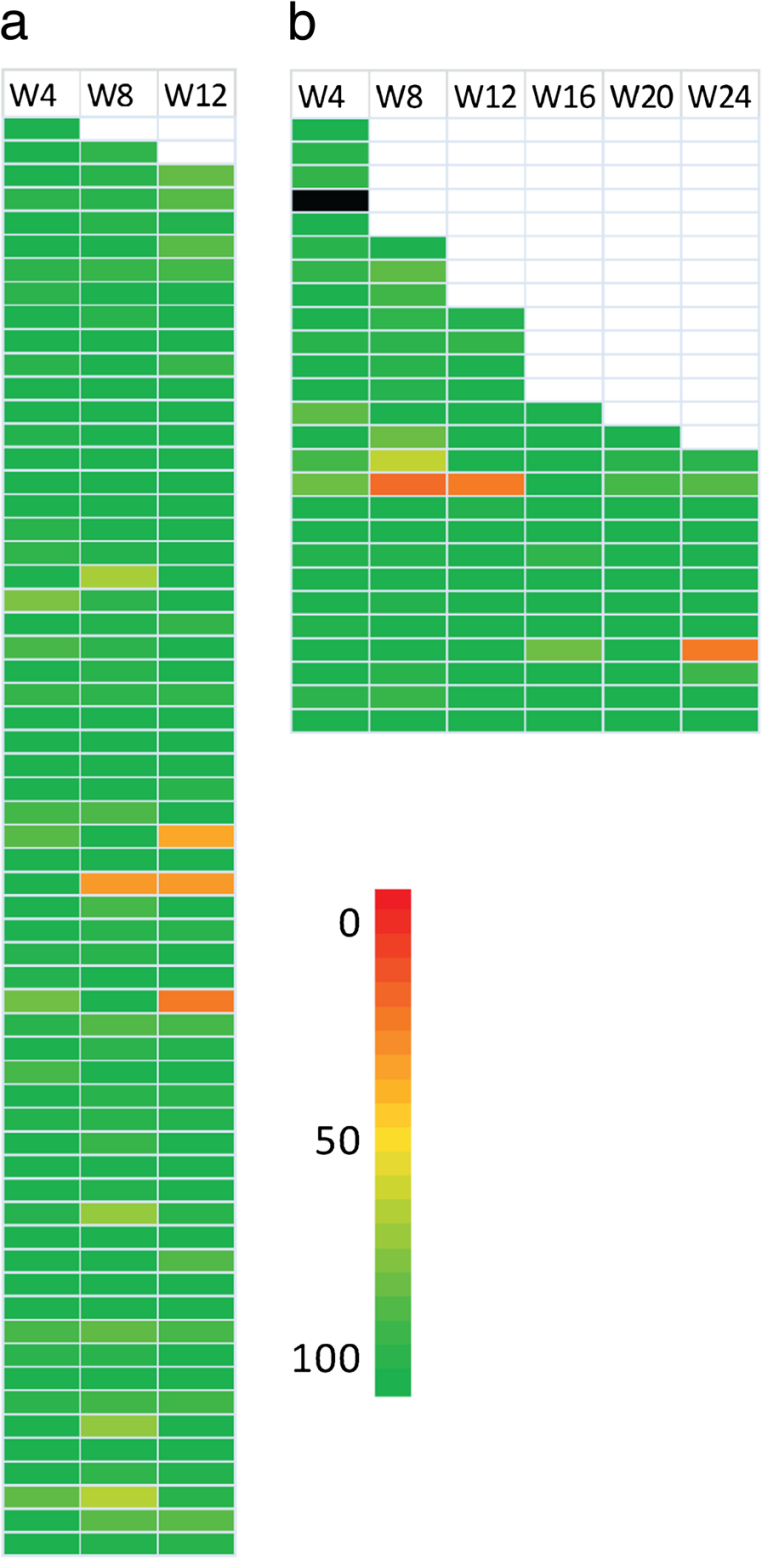
Four-weekly RBV adherence among HCV genotype 2/3 participants on shortened (12 weeks; *n* = 61; panel **a**) and standard therapy (24 weeks; *n* = 26; panel **b**). Each row represents a study participant. Colours represent each participant’s RBV adherence during each four-week period with dark green representing 100% adherence and red representing 0% adherence. White represents discontinuation before completion of the corresponding time-point while black represents missing data for both retuned pill counts and patient reported adherence before treatment discontinuation

Four-weekly RBV adherence among those receiving 12 weeks of therapy and 24 weeks of therapy is shown in Fig. 3. Returned pill counts were used in 276 of the 284 on treatment visits in Fig. 3 (97%). In the case of missing pill counts (*n* = 8) participant completed questionnaire data was used where available (*n* = 7). Data was not available, either from pill counts or patient completed questionnaires for one study visit. The mean overall on-treatment RBV adherence was similar between those receiving 12 and 24 weeks of therapy (94.8% vs. 94.1%, *P =* 0.751). The mean on-treatment adherence to RBV in the 12 week arm during weeks 1–4, 5–8, and 9–12 was 96.9%, 93.8% and 93.5% respectively. The mean on-treatment adherence to RBV in the 24 week arm during weeks 1–4, 5–8, and 9–12 was 96.6%, 88.3% and 95.2% respectively. Overall 65% of participants had >95% on-treatment RBV adherence.

### Factors associated with treatment completion and on-treatment RBV adherence

In unadjusted analyses, treatment completion occurred more frequently in those with recent injecting at baseline, recent heroin injecting at baseline, injecting weekly or more in the past month, and those in the 12 week arm but less often in people with recent amphetamine injecting at baseline and those who were >41 years of age (Table 3). The only factor that remained independently associated with treatment completion was shortened treatment arm (aOR 31.2, *P =* <0.001).

**Table 3:** Unadjusted potential predictors of treatment completion among the study population (*n* = 93)

	Number completed treatment (%; *n* = 71	Number not completing treatment (%; *n* = 22)	Unadjusted OR	95% CI	*P*
Age (years)					
≤ 41	38 (83)	8 (17)	1.00	-	-
> 41	33 (70)	14 (30)	0.50	0.19–1.33	0.164
Gender					
Female	13 (81)	3 (19)	1.00	-	-
Male	58 (75)	19 (25)	0.70	0.18–2.74	0.613
Education^a^					
< Tertiary	52 (79)	14 (21)	1.00	-	-
Tertiary or greater	18 (75)	6 (25)	0.81	0.27–2.42	0.703
Social functioning score					
< 17	31 (78)	9 (23)	1.00	-	-
≥ 17	40 (75)	13 (25)	0.89	0.34–2.36	0.820
Stable housing					
No	17 (77)	5 (23)	1.00	-	-
Yes	54 (76)	17 (24)	0.93	0.30–2.91	0.907
Current depression^b^					
No	30 (75)	10 (25)	1.00	-	-
Yes	37 (80)	9 (20)	1.37	0.49–3.80	0.545
Hazardous alcohol consumption^c^					
No	58 (81)	14 (19)	1.00	-	-
Yes	11 (83)	2 (15)	1.71	0.35–8.43	0.512
Current OST					
No	19 (70)	8 (30)	1.00	-	-
Yes	52 (79)	14 (21)	1.56	0.57–4.32	0.388
Injecting (last month)					
No	26 (68)	12 (32)	1.00	-	-
Yes	45 (82)	10 (18)	2.08	0.79–5.47	0.139
Frequency of injecting (last month)					
Never	26 (68)	12 (32)	1.00	-	-
Less than weekly	17 (77)	5 (23)	1.57	0.47–5.26	0.465
Weekly or greater	28 (85)	5 (15)	2.58	0.80–8.34	0.112
Heroin injecting (last month)					
No	43 (72)	17 (28)	1.00	-	-
Yes	28 (85)	5 (15)	2.21	0.73–6.68	0.159
Cocaine injecting (last month)					
No	63 (76)	20 (24)	1.00	-	-
Yes	8 (80)	2 (20)	1.27	0.25–6.48	0.774
Amphetamine injecting (last month)					
No	63 (80)	16 (20)	1.00	-	-
Yes	8 (57)	6 (43)	0.34	0.10–1.12	0.075
Injecting on therapy					
No	30 (83)	6 (17)	1.00	-	-
Yes	41 (79)	11 (21)	0.75	0.25–2.24	0.601
Therapy duration*					
24 weeks	13 (50)	13 (50)	1.00	-	-
12 weeks	58 (95)	3 (5)	19.33	4.81–77.78	<0.001

*Among participants who attended study week 4

^a^Missing data for three participants

^b^Missing data for seven participants

^c^Missing data for eight participants; percentages refer to row percentages

In unadjusted analyses, no factors were associated with >95% on-treatment RBV adherence (Table 4). In GEE analyses, later study visit was not associated with adherence to RBV (OR 1.00, *P =* 0.489).

**Table 4:** Unadjusted potential predictors of RBV adherence among the study population with available RBV adherence data (*n* = 86)

	RBV adherence of 95% (%; *n* = 59)	RBV adherence of < 95% (%; *n* = 27)	Unadjusted OR	95% CI	*P*
Age (years)					
≤ 41	29 (66)	15 (34)	1.00	-	-
> 41	30 (71)	12 (29)	1.25	0.50–3.11	0.632
Gender					
Female	49 (69)	22 (31)	1.00	-	-
Male	10 (67)	5 (33)	1.14	0.35–3.72	0.833
Education^a^					
< Tertiary	43 (69)	19 (31)	1.00	-	-
Tertiary or greater	15 (71)	6 (29)	1.18	0.40–3.48	0.766
Social functioning score					
< 17	29 (76)	9 (24)	1.00	-	-
≥ 17	30 (63)	18 (38)	0.53	0.21–1.38	0.195
Current depression^b^					
No	24 (65)	13 (35)	1.00	-	-
Yes	30 (68)	14 (32)	1.11	0.44–2.80	0.818
Hazardous alcohol consumption^c^					
No	48 (68)	23 (32)	1.00	-	-
Yes	8 (67)	4 (33)	0.94	0.26–3.44	0.924
Current OST					
No	16 (64)	9 (36)	1.00	-	-
Yes	43 (70)	18 (30)	1.26	0.48–3.36	0.638
Injecting (last month)					
No	22 (69)	10 (31)	1.00	-	-
Yes	37 (69)	17 (31)	0.95	0.37–2.42	0.908
Frequency of injecting (last month)					
Never	22 (69)	10 (31)	1.00	-	-
Less than weekly	14 (67)	7 (33)	0.87	0.27–2.81	0.815
Weekly or greater	23 (70)	10 (30)	1.00	0.35–2.86	1.000
Heroin injecting (last month)					
No	35 (66)	18 (34)	1.00	-	-
Yes	24 (73)	9 (27)	1.33	0.51–3.46	0.554
Cocaine injecting (last month)					
No	52 (68)	24 (32)	1.00	-	-
Yes	7 (70)	3 (30)	0.77	0.25–4.44	0.940
Amphetamine injecting (last month)					
No	51 (69)	23 (31)	1.00	-	-
Yes	8 (67)	4 (33)	0.88	0.24–3.24	0.853
Injecting on therapy					
No	25 (74)	9 (26)	1.00	-	-
Yes	34 (65)	18 (35)	0.65	0.25–1.69	0.380
Therapy duration					
24 weeks	18 (72)	7 (28)	1.00	-	-
12 weeks	41 (67)	20 (33)	0.80	0.29–2.22	0.664

^a^Missing data for three participants

^b^Missing data for five participants

^c^Missing data for three participants; percentages refer to row percentages

### Impact of missed doses of PEG-IFN and RBV, and treatment completion on SVR

In unadjusted analyses, SVR occurred more frequently among those who completed treatment (OR 34.4, *P* = <0.001). Neither 100% PEG-IFN adherence, nor >95% RBV adherence were associated with SVR in unadjusted analyses and were therefore not included in the adjusted model. In adjusted analyses, when adjusted for all factors found to be associated with SVR in the primary analysis [11], treatment completion remained as being associated with SVR (aOR 23.86, *P* = 0.003; Table 5).

**Table 5:** Unadjusted and adjusted models of adherence-related predictors of SVR among the study population

	SVR (%)	Unadjusted OR	95% CI	*P*	Adjusted OR	95% CI	*P*
95% ribavirin adherence*							
No	17 (63)	1.00	-	-	-	-	-
Yes	44 (73)	1.62	0.61–4.26	0.330	-	-	-
100% PEG-IFN adherence*							
No	58 (67)	1.00	-	-	-	-	-
Yes	3 (50)	2.00	0.38–10.53	0.413	-	-	-
Completed therapy							
No	2 (9)	1.00	-	-	1.00	-	-
Yes	59 (83)	34.41	6.90–171.55	0.000	23.86	2.94–193.84	0.003

The adjusted model is adjusted for all factors associated with SVR from the primary analysis [11]

*On-treatment adherence

## Discussion

This study investigated treatment completion and the adherence to response-guided directly observed PEG-IFN and self-administered ribavirin treatment for chronic HCV genotypes 2/3 among PWID with ongoing drug use and those receiving OST. The results demonstrated high adherence to directly observed PEG-IFN and self-administered RBV therapy, particularly among participants receiving 12 weeks, as opposed to 24 weeks, of therapy. Being scheduled to receive 12 weeks of therapy was an independent predictor of treatment completion. There were no independent predictors of >95% RBV adherence in the population. Neither recent injection drug use prior to treatment, nor injection drug use while on treatment, were associated with treatment completion or >95% on-treatment RBV adherence. Further, the majority of the sub-optimal treatment exposure was due to early discontinuation of therapy rather than missed doses while on therapy. Finally, while neither PEG-IFN nor RBV adherence was an independent predictor of SVR, treatment completion was found to be an independent predictor of SVR. These data have important clinical implications informing HCV management among PWID with ongoing drug use and those receiving OST in the DAA era, given that the majority of licensed regimens require only 12 weeks of therapy.

Overall, adherence to PEG-IFN/RBV therapy was high with few participants (6%) missing ≥1 dose of PEG-IFN while on therapy, with no one missing more than one dose for a high on-treatment adherence of 98%. This is consistent with previous studies of PEG-IFN adherence where on-treatment adherence ranged from 74% to 99% [3, 14–17]. While slightly lower than PEG-IFN adherence, a similarly high on-treatment RBV adherence was observed (94.6%) with 71% of participants taking at least 95% of their RBV doses. The proportion of the population with >95% adherence is lower than was reported in the C-EDGE CO-STAR trial (96%), a randomized controlled trial of elbasvir-grazoprevir in people receiving stable opioid agonist therapy [18]. This is potentially due to the higher toxicity of PEG-IFN/RBV therapy or the increased pill burden due to the inclusion of RBV. Also, the C-EDGE CO-STAR trial likely represents a more stable population given the inclusion of only those on stable OST and the exclusion of those actively using drugs of potential abuse. As such, it is difficult to directly compare these results.

Participants who were allocated 12 weeks of therapy demonstrated a higher proportion completing therapy than those allocated 24 weeks of therapy. Recent injection drug use prior to and during treatment was not associated with PEG-IFN adherence, RBV adherence, or treatment completion, consistent with previous data [3, 10, 19–22].

While PEG-IFN based therapies have recently been replaced by new DAA therapies in many settings, data on the adherence to HCV therapies among PWID is still needed. In many countries, concerns still exist regarding the adherence to therapy among active PWID [10, 23]. As a result many countries have restricted the use of DAA therapies to those without current injecting [23–25]. A better understanding of adherence among active injectors is therefore needed to inform policy and remove the restrictions placed on DAA therapies. This data also highlights the positive effect of shortened therapy on treatment completion in this population as pushes to further shorten HCV therapy continue. This study includes data on adherence to self-administered ribavirin, providing some insight into adherence of an oral twice-daily antiviral therapy. Given the increased tolerability and simplicity DAA regimens compared to PEG-IFN and ribavirin-based therapies, adherence should be comparable, if not better, than that observed with twice-daily ribavirin, Further data is needed to assess adherence to DAA-based therapy among people with ongoing injecting drug use.

There are some limitations to this study. Adherence to RBV was determined based on returned pill counts where available and patient surveys where pill counts were unavailable. While pill counts are generally a better estimate of the true adherence as compared to patient reported adherence [26], there is still the potential for overestimation through lost pills. The measurement of adherence to PEG-IFN was more robust, given that this was a directly observed dose in the presence of the study nurse who recorded when the dose was taken. Further, adherence is a very complex phenomenon and may be influenced by a number of unmeasured factors (e.g. past experience with adherence to other medications, patient-doctor relationships). Lastly, the small sample size of this study is a limitation. With a larger sample size, it would be possible to more accurately estimate the true effects of various factors on HCV treatment adherence and the true effect of treatment adherence on SVR. In addition, while the international nature of this study increases the generalizability to globally diverse PWID populations, the participants recruited into this study may represent a somewhat selected group, based on improved engagement in care. Irrespective of these limitations, this is the first international study to evaluate adherence to HCV therapy among PWID and those receiving OST.

## Conclusions

This study demonstrates high adherence to response-guided directly observed pegylated-interferon and self-administered ribavirin therapy for chronic HCV genotypes 2/3 infection among people with ongoing injecting drug use or receiving OST. These data suggest that shortening HCV therapy has the potential to increase treatment completion among PWID. This is of particular importance given the interest in evaluating shorter durations of DAA therapy. Further, injecting drug use both prior to and during treatment was not associated with reduced adherence to therapy or treatment completions. These data suggest that adherence to HCV therapy among HCV infected PWID is not compromised by ongoing injection drug use and supports guidelines which suggest that active PWID should not be excluded from therapy; rather decisions should be made on a case-by-case basis [27–30]. Further studies are needed to characterize adherence to interferon-free DAA therapies among PWID and those receiving OST to better understand whether adherence is actually a problem in this population in the DAA era.

## Additional file

Additional file 1: Local ethics committees. (DOCX 16 kb)

## Abbreviations

aOR: Adjusted odds ratio
DAA: Direct acting antiviral
HCV: Hepatitis C virus
HIV: Human immunodeficiency virus
OR: Odds ratio
OST: Opioid substitution therapy
PEG-IFN: Pegylated-interferon alfa-2b
PWID: People who inject drugs
RBV: Ribavirin
RVR: Rapid virological response
SVR: Sustained virological response
