# Supplementary material for: Adherence to response-guided pegylated interferon and ribavirin for people who inject drugs with hepatitis C virus genotype 2/3 infection: the ACTIVATE study
Source: BMC Infect Dis. 2017 Jun 13;17:420. doi: 10.1186/s12879-017-2517-3 (PMC5470219; doi:10.1186/s12879-017-2517-3)
Supplement: Additional file 1: — Local ethics committees. (DOCX 16 kb) [file 12879_2017_2517_MOESM1_ESM.docx]

**Appendix: Local ethics committees**

1. St Vincent's Hospital Sydney Human Research Ethics Committee (Sites: St Vincent’s Hospital, John Hunter Hospital, Nepean Hospital) – ref number: HREC/11/SVH/7
2. Royal Adelaide Hospital Research Ethics Committee (Site: Royal Adelaide Hospital) – ref number: R20110720
3. The Alfred Hospital Ethics Committee (Site: Alfred Hospital) – ref number: 429/11
4. Institutional Review Board Services (Site: Vancouver Infectious Diseases Centre) – ref number: Pro00014060
5. Toronto East General Hospital Research Ethics Board (Site: South Riverdale Community Health Centre) – ref number: 482-1110-InD-010
6. Comite d’Ethique de la recherche du CHUM (Site: Centre Hospitalier de l’Universite de Montreal) – ref number: 2012-3134
7. Commissie voor Medische Ethiek ZNA, Antwerp (Site: ZNA Stuivenberg Antwerp) – ref number: BE-80-1120017
8. Commissie Medische Ethiek Ziekenhuis Oost Limburg (Site: Ziekenhuis Oost Limburg (ZOL), Genk) – ref number: 12032/U
9. Ludwig Maximilians Universitat Ethikkommission (Site: PIT, Munich) – ref number: 319-12
10. Regional komitter for medisinsk og helsefaglig forskningsetikk (Site: AHUS, Oslo) – ref number: 2011/2014/REK sør-øst A
11. Kantonale Ethikkommission Bern (Site: Inselspital, Bern) – ref number: 139/12
12. Kantonale Ethikkommission Zurich (Site: ARUD, Zurich) – ref number: 2012-0024
13. Ethikkommission Nordwest und Zentralschweiz (Site: Zentrum für Suchtmedizin, Basel) – ref number: 22/12
14. NRES Committee East Midlands - Derby (Sites: Tower Hamlets Specialist Addiction Unit East London, Nottingham University Hospitals NHS Trust) – ref number: 12/EM/0010
